# Multiple Targets of Salicylic Acid and Its Derivatives in Plants and Animals

**DOI:** 10.3389/fimmu.2016.00206

**Published:** 2016-05-26

**Authors:** Daniel F. Klessig, Miaoying Tian, Hyong Woo Choi

**Affiliations:** ^1^Boyce Thompson Institute, Cornell University, Ithaca, NY, USA; ^2^Department of Plant and Environmental Protection Sciences, University of Hawaii at Manoa, Honolulu, HI, USA

**Keywords:** salicylic acid, salicylic acid-binding proteins, salicylic acid derivatives, plant immunity, animal immunity and inflammation, disease, common plant and animal targets

## Abstract

Salicylic acid (SA) is a critical plant hormone that is involved in many processes, including seed germination, root initiation, stomatal closure, floral induction, thermogenesis, and response to abiotic and biotic stresses. Its central role in plant immunity, although extensively studied, is still only partially understood. Classical biochemical approaches and, more recently, genome-wide high-throughput screens have identified more than two dozen plant SA-binding proteins (SABPs), as well as multiple candidates that have yet to be characterized. Some of these proteins bind SA with high affinity, while the affinity of others exhibit is low. Given that SA levels vary greatly even within a particular plant species depending on subcellular location, tissue type, developmental stage, and with respect to both time and location after an environmental stimulus such as infection, the presence of SABPs exhibiting a wide range of affinities for SA may provide great flexibility and multiple mechanisms through which SA can act. SA and its derivatives, both natural and synthetic, also have multiple targets in animals/humans. Interestingly, many of these proteins, like their plant counterparts, are associated with immunity or disease development. Two recently identified SABPs, high mobility group box protein and glyceraldehyde 3-phosphate dehydrogenase, are critical proteins that not only serve key structural or metabolic functions but also play prominent roles in disease responses in both kingdoms.

## Introduction

In plants, salicylic acid (SA) was viewed as a relatively unimportant secondary metabolite until the late twentieth century, when Raskin and coworkers revealed its involvement in signaling thermogenesis (1) and our group (2), together with Métraux and colleagues (3), demonstrated its importance in activating disease resistance. Today, a Google search for the “number of papers on salicylic acid and plant disease resistance” lists ~59,000. Many, if not most, of these studies confirm SA’s central role in immunity, principally against biotrophic and hemibiotrophic pathogens.

**Figure 1:**
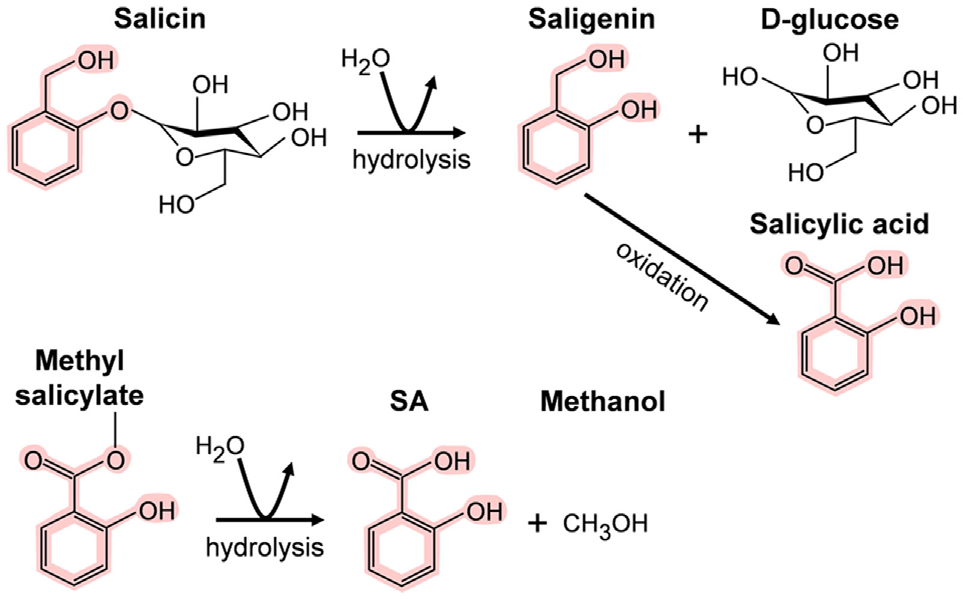
**Metabolism of salicin and methyl salicylate to salicylic acid (SA)**. The SA core is highlighted in pink.

In contrast, the importance of SA and its derivatives (collectively called salicylates) as pharmacological agents has long been appreciated. Salicin, the SA derivative that is the active ingredient in willow bark, was isolated in 1828; however, Hippocrates, the father of medicine, reportedly prescribed willow bark to reduce fever and the pain of childbirth in the fifth millennium B.C. High levels of salicylates have been detected in several plant species besides willow. For example, meadowsweet contains both salicin and methyl salicylate (MeSA), another medicinal derivative that also is known as the highly fragrant oil of wintergreen. In animals/humans, these “prodrugs” are converted to SA upon digestion (Figure 1) (4, 5). The sources and chemical structures of several useful natural salicylates are shown in Table 1. Not only have medicinal plants rich in salicylates been used worldwide in many different cultures for thousands of years but also they continue to be used today. In this regard, the most famous SA derivative, acetyl SA, is a relative “new comer” as it was first synthesized by Bayer and Company in 1897 and subsequently sold under the trade name aspirin. Interestingly, the name SA is derived from the Latin name for white willow (*Salix alba*), while the term aspirin is derived from meadowsweet (*Spiraea ulmaria*).

**Table 1:** **List of useful salicylates present in various plants**.

Salicylates	Structure	Plant source	Use	Reference
Salicin^a^	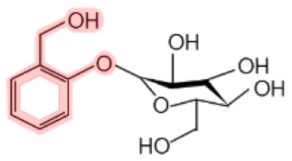	Aspen (*Populus tremula*) Black haw (*Viburnum prunifolium*) White willow (*Salix alba*) Meadowsweet (*Spiraea ulmaria*)	Analgesic, antipyretic, anti-inflammatory	(6–9)
Methyl salicylate^a^	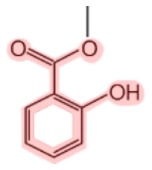	Birch tree (*Betula lenta*) Mango (*Mangifera indica*) Meadowsweet (*Spiraea ulmaria*) Wintergreen (*Gaultheria rocumbens*) Guelder-rose (*Viburnum opulus*)	Analgesic (joint and muscular pain), fragrance	(9–13)
Amorfrutins^b^	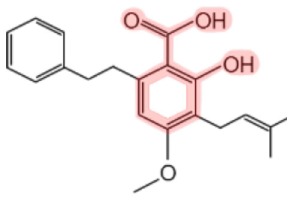	Indigo bush (*Amorpha fruticosa*) Licorice (*Glycyrrhiza foetida*)	Antidiabetic, anti-cancer, anti-inflammatory	(14–18)
Benzyl salicylate	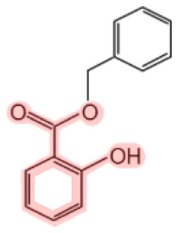	Cananga tree (*Cananga odorata*) Mango (*Mangifera indica*) Wintergreen (*Gaultheria rocumbens*)	Fragrance, UV light absorber	(11, 12, 19, 20)
*Cis*-3-hexenyl salicylate	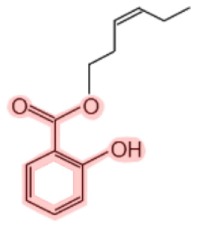	Mango (*Mangifera indica*) Wintergreen (*Gaultheria rocumbens*)	Fragrance	(11, 12)
4-Hepten-2-yl salicylate	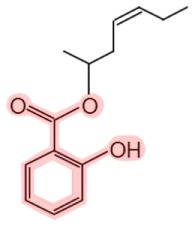	Ashoka (*Saraca indica*)	Fragrance	(21, 22)
Isoamyl salicylate	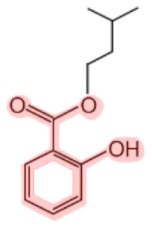	Wintergreen (*Gaultheria rocumbens*)	Fragrance	(12)

*The salicylic acid core is highlighted in pink*.

*^a^Salicin and methyl salicylate are converted into SA (see Figure 1 for details)*.

*^b^Armofrutin 1 is shown as a representative structure of various amorfrutins*.

## One or a Few Receptors vs. Multiple Targets

The current view is that hormones in plants, as well as in animals, exert their effect(s) by binding to one or a small number of receptors. Is SA’s mechanism(s) of action consistent with this dogma? The answer to this question is currently unclear. One or more members of the non-expressor of pathogenesis-related protein (NPR) family were proposed to function as SA receptors in plants (23, 24). The molecular mechanism(s) through which SA mediates NPR1’s function as a co-activator of immunity-induced transcriptional reprograming is currently unresolved. While the identification of NPR proteins as SA targets is a major step toward elucidating SA’s mechanisms of action in defense against microbial pathogens, the upregulation of some plant immune responses, including expression of a subset of defense-related genes, is mediated *via* a pathway(s) that is dependent on SA, but independent of NPR1 (25, 26). Moreover, it is not known whether NPR1/NPR3/NPR4 is involved in mediating SA’s effects on other plant processes, including growth and development and/or response to abiotic stress (27). Thus, NPR proteins may not function as SA receptors in the traditional sense.

The identification of almost 30 SA-binding proteins (SABPs) using traditional purification approaches (28–32) and genome-wide, high-throughput screens (33, 34) (http://bioinfo.bti.cornell.edu/SA2010/), further argues that SA exerts its effects *via* more than one or a few receptors. Given that SA-binding alters the activity of many of these SABPs, several difficult questions must be considered: should all identified SABPs listed in Table 2 be promoted to SA receptor status? Alternatively, should the level of SA-binding affinity be used as a criterion, with only those SABPs displaying high affinity qualifying for receptor status? The latter scenario presents additional concerns, as it is unclear what dissociation constant (*K*_d_) value should serve as the cutoff, and who should decide it? In addition, the affinities of the reported NPR receptors overlap those of several SABPs. For example, the MeSA esterase SABP2 from tobacco and its *Arabidopsis* ortholog MES9 have high affinities for SA (apparent *K*_d_ = 0.092 and ~0.200 μM, respectively) (31, 32, 35), which are similar to those of NPR1 (*K*_d_ = 0.140–0.190 μM) (24, 34) and NPR4 (*K*_d_ = 0.046 μM) (23). By contrast, the SA affinity displayed by NPR3, the other reported SA receptor, is considerably lower (*K*_d_ = 1 μM) and approaches those of catalase (*K*_d_ = 15.5 μM) (36) and carbonic anhydrase (*K*_d_ = 3.7 μM) (30). We propose that proteins which bind hormones (or other ligands) and as a result have altered function or activity be termed “targets” of their corresponding hormone. The term receptor could be applied to a subset of these targets that meet additional criteria. For example, classic receptors for water-soluble hormones, which cannot diffuse through the plasma membrane, span this membrane in order to detect extracellular hormones at the cell surface and initiate downstream intracellular signaling (e.g., G protein-coupled receptors and the enzyme-linked receptors) (37, 38). On the other hands, many receptors for steroids, which readily diffuse through the plasma membrane, are located intracellularly, and directly regulate gene transcription upon complex formation with their cognate hormone (39). Given the many targets through which SA appears to mediate its effects on diverse physiological and pathological plant processes, we suggest that this represents a paradigm shift for how, at least, some hormones function. Furthermore, this novel paradigm may prove applicable to other plant hormones and perhaps even some animal hormones.

**Table 2:** **List of plant SA-binding proteins (SABPs)**.

Protein name	Plant species	Genetic locus of *Arabidopsis* SABPs	Interaction with SA plays a role in plant immunity	Reference
Catalase	Tobacco		Yes	(28)
Ascorbate peroxidase	Tobacco		Yes	(29)
Methyl salicylate esterases (tobacco SABP2 and *Arabidopsis* AtMES1, 2, 4, 7, and 9)	Tobacco, *Arabidopsis*	At2g23620	Yes	(31, 32)
		At2g23600		
		At2g23580		
		At2g23560		
		At4g37150		
Carbonic anhydrase (SABP3)	Tobacco, *Arabidopsis*	At3g01500	Yes	(30, 40)
NPR1	*Arabidopsis*	At1G64280	Yes	(24, 34)
NPR3	*Arabidopsis*	At5G45110	Yes	(23)
NPR4	*Arabidopsis*	At4G19660	Yes	(23)
Glutathione *S*-transferases PHI (GSTF2, 8, 10, and 11)	*Arabidopsis*	At4g02520		(33)
		At2g47730		
		At2g30870		
		At3903190		
Ketoglutarate dehydrogenase E2 subunit (KGDHE2)	*Arabidopsis*, tomato	At5g55070	Yes	(33, 41)
Thimet oligopeptidases (TOP1 and 2)	*Arabidopsis*	At5g65620	Yes	(42)
		At5g10540		
Glyceraldehyde 3-phosphate dehydrogenases (GAPDHA1, A2, C1, and C2)	*Arabidopsis*	At3g26650	Yes	(43)
		At1g12900		
		At3g04120		
		At1g13440		
Thioredoxin M-type 1 (TRX-m1)	*Arabidopsis*	At1g03680		(34)
Tripeptidyl peptidase II (TPP2)	*Arabidopsis*	At4g20850		(34)
Serine hydroxyl methyltransferase 4 (SHM4)	*Arabidopsis*	At4g13930		(34)
Lipoxygenase 2 (LOX2)	*Arabidopsis*	At3g45140		(34)
Glutathione peroxidase (GPX2)	*Arabidopsis*	At2g31570		(34)
Glutamine synthetase (GSR2)	*Arabidopsis*	At1g66200		(34)
Hydroxypyruvate 2 (HPR2)	*Arabidopsis*	At1g79870		(34)
Ribulose bisphosphate carboxylase small subunit 1A (RBCS1A)	*Arabidopsis*	At1g67090		(34)
UDP-d-glucose/UDP-d-galactose 4-epimerase 2 (UGE2)	*Arabidopsis*	At4g23920		(34)
High mobility group B3 (HMGB3)	*Arabidopsis*	At1g20696	Yes	(44)

Of the various SABPs whose SA-binding affinities have been determined, their *K*_d_ values span from 0.046 to 15.5 μM. Consistent with this 300-fold range, SA levels in plants can vary dramatically (Table 3). Not only do they differ between various plant species but they also can vary within an individual plant depending on the tissue type, subcellular compartment, and developmental stage. In addition, SA levels can vary with respect to the time and/or location after reception of an (a)biotic stress, such as pathogen infection (Table 3). Thus, we hypothesize that SA exerts its multitudinous effects by differentially interacting with various SABPs depending on their affinity for SA, their location, and the local SA concentration. Our analyses of SABP2, a MeSA esterase, and its role in signaling systemic defense responses in tobacco are consistent with this mechanism (45). Following pathogen infection, SA levels increase dramatically in the inoculated leaves, where much of it is converted to biologically inactive MeSA by SA/benzoic acid methyl transferase; once the SA concentration becomes sufficiently high, it binds in the active site of SABP2 and inhibits SABP2’s ability to convert MeSA back into SA (45). The resultant increase in MeSA facilitates its translocation to the distal, uninfected tissue. Since SA levels in the distal tissue are too low to inhibit SABP2, the transported MeSA is converted to active SA, which then induces and/or primes various systemic defense responses. Similarly, the interplay between SA, NPR1, and NPR3/4 fine-tunes NPR1 homeostasis in a SA concentration-dependent manner, which determines the levels and types of plant defense responses during pathogen infection (23, 27). The presence of SABPs exhibiting a wide range of affinities for SA, combined with the varying SA levels found in specific subcellular compartments, in different tissues, at different developmental stages, or during responses to environmental cues, provides tremendous flexibility and multiple mechanisms through which SA can exert its effects. Unfortunately, little is known about the concentrations and distributions of SA at the cellular and/or subcellular levels, because measurements are generally made on total tissue extracts. Thus, there is a pressing need for novel *in vivo* detection methods of SA (e.g., fluorescent probes) (46, 47) to provide a greater, more detailed understanding of SA functions in mediating the activities of its target or receptor proteins.

**Table 3:** **Endogenous salicylic acid levels in different plants**.

Plant	Sample (treatment)	SA (free)	Conjugated SA^a^	Reference
Cucumber (*Cucumis sativus*)	Leaf (No)	~0.04 μg/g FW	–	(3, 48)
	Leaf (*P.l*.)	~0.9 μg/g FW	~8.0 μg/g FW	
	Systemic leaf (*P.l*.)	~0.32 μg/g FW	~3.0 μg/g FW	
	Leaf (TNV)	~0.125 μg/g FW	~0.75 μg/g FW	
	Systemic leaf (TNV)	~0.1 μg/g FW	~0.1 μg/g FW	
	Phloem sap (No)	~0.1 μg/mL	–	
	Phloem sap (*C.l*.)	~0.9 μg/mL	–	
	Phloem sap (TNV)	~0.4 μg/mL	–	
Tobacco (*Nicotiana tabacum*)	Leaf (No)	0.05–0.3 μg/g FW	0.02–0.1 μg/g FW	(2, 49–51)
	Leaf (TMV)	2.0–20.0 μg/g FW^b^	1–75 μg/g FW	
	Systemic leaf (TMV)	~1.5 μg/g FW	~1.5 μg/g FW	
	Phloem sap (No)	<0.01 μg/mL	<0.01 μg/mL	
	Phloem sap (TMV)	~0.25 μg/mL	–	
Rice (*Oryza sativa*)	Leaf (No)	~10 μg/g FW^c^	–	(52)
	Leaf (*P.s*.)	~10 μg/g FW	–	
*Arabidopsis* (*Arabidopsis thaliana*)	Leaf (No)	0.07–1.0 μg/g FW	0.15–4.0 μg/g FW	(53–56)
	Leaf (*P.s*.)	1.5–3.0 μg/g FW	5.0–8.0 μg/g FW	
	Systemic leaf (*P.s*.)	~0.2 μg/g FW	~0.6 μg/g FW	
Potato (*Solanum tuberosum*)	Leaf (No)	0.2–2 μg/g FW	5.0–15.0 μg/g FW	(57–59)
	Leaf (A.a.)	8–10 μg/g FW	~4 μg/g FW	
	Stem (No)	~1 μg/g FW^d^	~1.5 μg/g FW	
Pepper (*Capsicum annuum*)	Leaf (No)	~0.2 μg/g FW	~0.8 μg/g FW	(60)
	Leaf (*X.c*.)	~2 μg/g FW	~2 μg/g FW	

*^a^SA conjugated to glucose as SA 2-O-β-d-glucoside (SAG) or salicylate glucose ester (SGE) (61)*.

*^b^See figure 2 in Enyedi et al. (49) for distribution of endogenous SA around necrotic lesions induced by TMV*.

*^c^Free SA level varies among different varieties of rice plants (52)*.

*^d^See figure 1 and 4 in Navarrea and Mayoa (58) for endogenous SA levels in different potato organs and in potato plants during different seasons, respectively*.

*No, no treatment; P.l., Pseudomonas lachrymans; TNV, tobacco necrosis virus; C. l., Colletotrichum lagenarium; P.s., Pseudomonas syringae; TMV, tobacco mosaic virus; A.a., arachidonic acid; X.c., Xanthomonas campestris; FW, fresh weight*.

## Multiple Targets of SA and Its Derivatives in Humans

**Figure 2:**
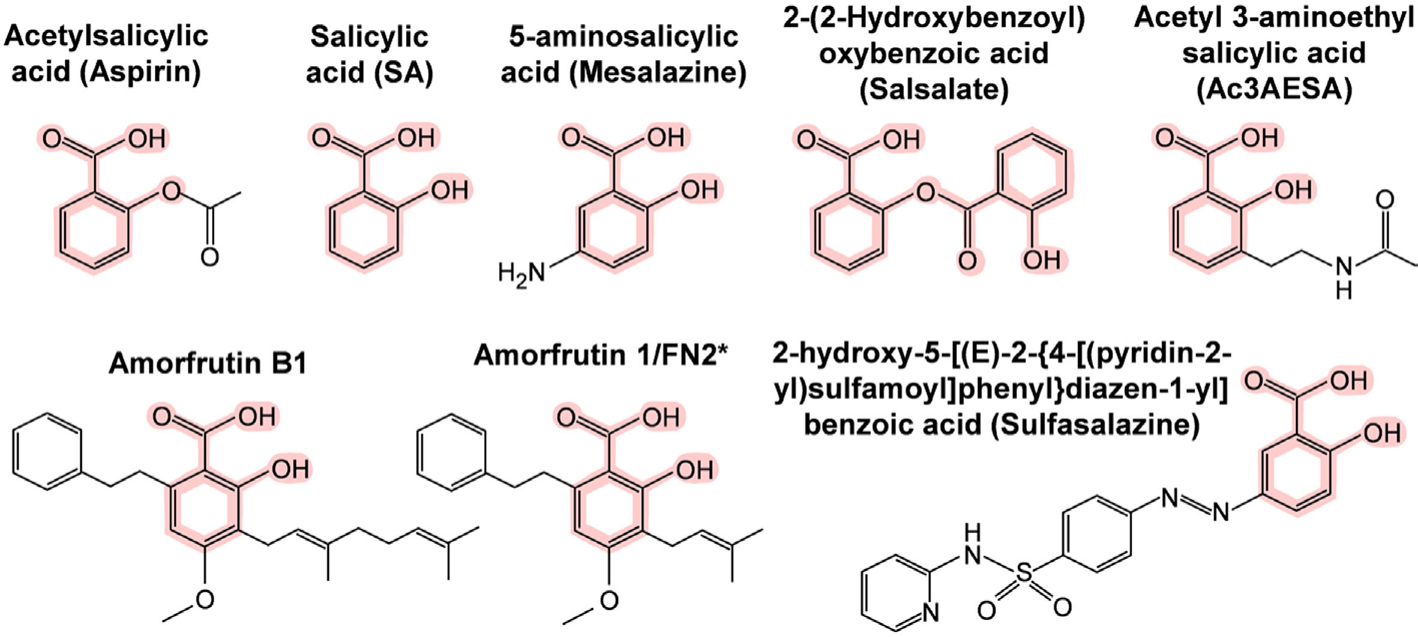
**Chemical structures of SA and its synthetic and natural derivatives**. The SA core is highlighted in pink. *This amorfrutin was called amorfrutin 1 in Weidner et al. (17) and FN2 in Choi et al. (14).

The first SA targets identified in humans were the cyclooxygenases COX1 and COX2. These enzymes convert arachidonic acid, the major plasma membrane fatty acid in animals, into prostaglandins. Prostaglandins have hormone-like activities that induce pain, inflammation, swelling, and fever. Notably, these are the same symptoms that are relieved by ingestion of salicylate-rich medicinal plants, SA, or acetyl SA (aspirin). In the early 1970s, Vane and coworkers discovered that aspirin irreversibly inhibits COX1 and COX2 by acetylating a serine near the active site, which prevents access of the arachidonic acid substrate to the active site (62, 63). This hallmark discovery has dominated the field ever since and supports the prevailing view in the biomedical community of how aspirin works. However, this hypothesis cannot explain how salicylate-rich medicinal plants, which have been used worldwide for millennia, and SA, which was used extensively for half a century before the synthesis of aspirin, are able to treat pain, inflammation, and fever. Naturally occurring salicylates and SA are only weak inhibitors of COX1 and COX2, as they cannot acetylate them (63), and yet SA has most of the same pharmacological effects as aspirin. Moreover, aspirin is rapidly converted to SA in the human body with a half-life of about 20 min (64, 65). In contrast, plasma SA levels after aspirin ingestion rapidly increase and are sustained for more than 12 h (66). These facts argue that there must be additional SA targets besides the cyclooxygenases. During the past three decades, 15 additional potential targets of aspirin, SA, and/or SA prodrugs (Figure 2) have been identified (Table 4). Several of these SA/aspirin targets are associated with inflammation, including tumor necrosis factor alpha (TNFα), nuclear factor-kappa-B (NF-κB), inhibitor of NF-κB kinase subunit beta (Iκκ-β), and high mobility group box 1 (HMGB1), while others regulate energy metabolism, such as adenosine monophosphate-activated protein kinase (AMPK) and peroxisome proliferator-activated receptor gamma (PPARγ). For example, sulfasalazine (see Figure 2 for its structure) blocks TNFα-induced T-cell activation by inhibiting the binding of TNFα to its receptor (67). Aspirin and sodium salicylate are proposed to inhibit transcription factor NF-κB-mediated pro-inflammatory signaling by inhibiting Iκκ-β kinase activity, which induces degradation of inhibitory protein of NF-κB (IκB) by phosphorylation (68, 69). Unfortunately, the levels of aspirin or SA needed to alter the activities of many of these potential targets are very high and are likely to have toxic side effects in humans.

**Table 4:** **List of human proteins targeted by salicylates**.

Protein name	Salicylate	Reference
Arachidonate 5-lipoxygenase (ALOX5)	5-aminosalicylic acid, sulfasalazine	(70)
Tumor necrosis factor alpha (TNFα)	Sulfasalazine	(67)
Cyclooxygenase-1 (COX-1)	Aspirin	(63, 71)
Cyclooxygenase-2 (COX-2)	Aspirin, sodium salicylate	(63, 71, 72)
Nuclear factor-kappaB (NF-κB)	Aspirin, sodium salicylate	(69)
Cathepsin A (CTSA)	Aspirin	(73)
Inhibitor of nuclear factor-kappa-B kinase subunit beta (Iκκ-β)	Aspirin, sodium salicylate	(68)
Ribosomal S6 kinase 2 (RSK2)	Aspirin, salicylic acid	(74)
Adenosine monophosphate-activated protein kinase (AMPK)	Sodium salicylate	(75)
Peroxisome proliferator-activated receptor gamma (PPARγ)	Amorfrutins	(17)
Ferrochelatase (FECH)	Salicylic acid	(76)
Acetyltransferase p300 (P300)	Salsalate, salicylate	(77)
Glyceraldehyde 3-phosphate dehydrogenase (GAPDH)	Salicylic acid, amorfrutins B1 and FN2, acetyl 3-aminoethyl salicylic acid, 5-aminosalicylic acid	(14)
High mobility group box 1 (HMGB1)	Salicylic acid, amorfrutin B1, acetyl 3-aminoethyl salicylic acid	(15)
Cyclin-dependent kinase 2 (CDK2)	Aspirin, salicylic acid	(78)

## Plants and Animals Share Several SA Targets

**Figure 3:**
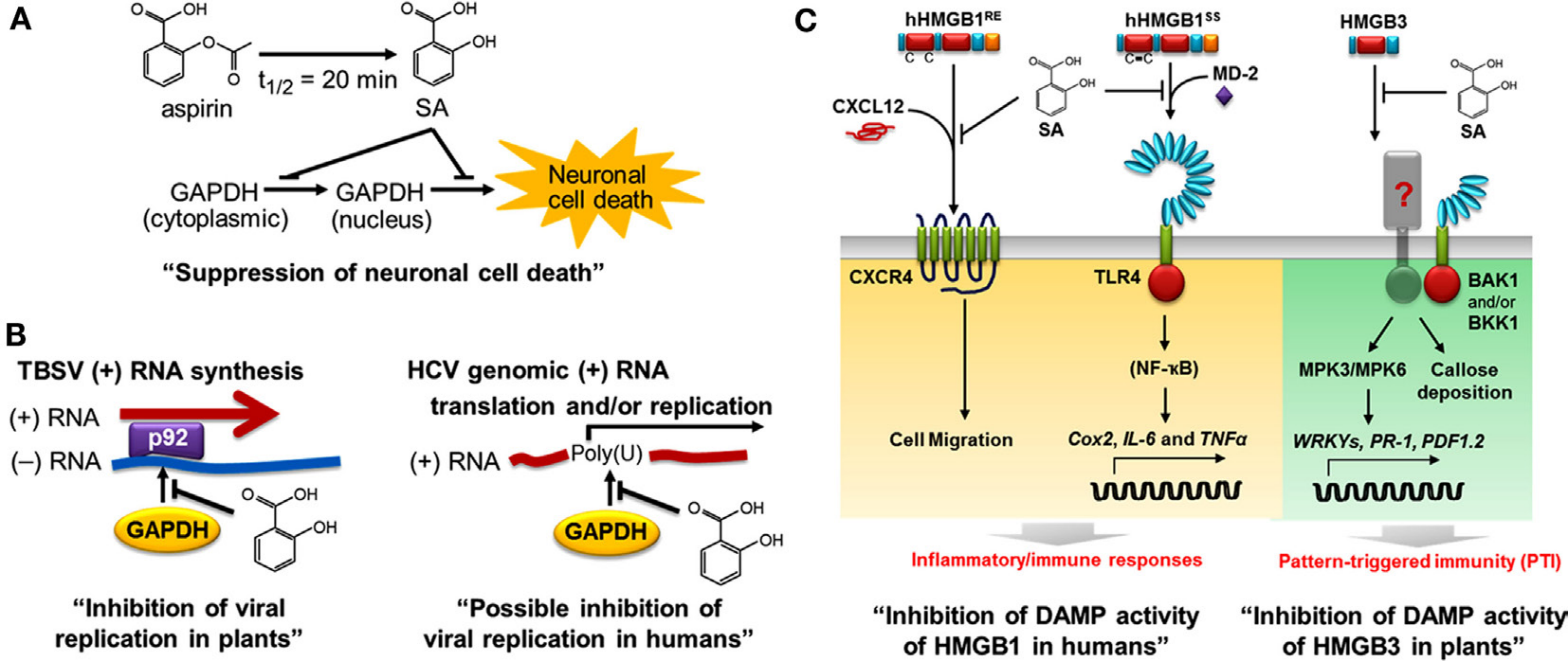
**Salicylic acid (SA) affects both plant and human health, in part through common targets such as GAPDH and HMGB proteins**. In plants, SA is a key hormone that modulates immune responses; in humans, it is the major metabolite of aspirin. **(A)** SA binds to human GAPDH and suppresses its translocation from cytoplasm to nucleus and the resulting cell death (14). **(B)** SA binds to GAPDH and suppresses its participation in viral replication. In plants, GAPDH binding to the minus (−) RNA strand of tomato bush stunt virus (TBSV) promotes plus (+) RNA strand synthesis by the viral RNA-dependent RNA polymerase p92 (82). SA inhibits the interaction between plant GAPDH and the (−) RNA strand of TBSV, thereby reducing viral replication (left panel) (43). In humans, Petrik et al. (83) reported that human GAPDH binds to the poly (U) tract of genomic hepatitis C virus (HCV) RNA, while SA and aspirin were subsequently shown to suppress HCV replication (84, 85). We found that SA inhibits human GAPDH binding to poly (U), suggesting that SA has a similar mechanism of action for inhibition of HCV and TBSV (right panel, Tian and Klessig, unpublished results). **(C)** SA inhibits the DAMP activities of HMGBs in humans (left panel) (15) and in plants (right panel) (44). Extracellular human HMGB1 functions as a damage-associated molecular pattern (DAMP or alarmin). SA binds to HMGB1, thereby inhibiting the pro-inflammatory activities of reduced and disulfide-bonded HMGB1 (hHMGB1^RE^ and hHMGB1^SS^, respectively). C–X–C chemokine receptor 4 (CXCR4) recognizes the heterocomplex of hHMGB1^RE^ and C–X–C motif-containing chemokine 12 (CXCL12) to induce cell migration, while the toll-like receptor 4 (TLR4) binds the heterocomplex of hHMGB1^SS^ and myeloid differentiation factor 2 (MD-2) (86) to activate expression of *Cox2* and pro-inflammatory cytokine genes (*IL-6* and *TNF*α). SA blocks these pro-inflammatory pathways (15). In plants, HMGB3 functions as a DAMP. Extracellular HMGB3 activates pattern-triggered immunity responses, including MAPK activation (MPK3 and MPK6), defense-related gene expression (WRKYs, *PR-1*, and *PDF1.2*), and callose deposition. The regulatory receptor-like kinases BAK1 and/or BKK1 are required for HMGB3 signaling through a yet to be discovered receptor. This figure is modified from Klessig (87).

Using a high-throughput screen, we recently identified several members of the *Arabidopsis* glyceraldehyde 3-phosphate dehydrogenase (GAPDH) family, including GAPDHC1, as SABPs. In both plants and animals, GAPDH plays a central role in glycolysis; in addition, some family members are usurped by invading viruses to facilitate their replication. For example, efficient replication of tomato bushy stunt virus (TBSV) requires binding of GAPDH to the 3′ end of the negative-strand RNA template for synthesis of the positive strand, which is translated or packaged into the virion (79). In collaboration with Peter Nagy’s group, we showed that SA inhibits TBSV replication by binding to GAPDH and thereby preventing its binding to the negative-strand RNA template in the replication complex (Figure 3) (43). Similarly, SA binding to human GAPDH suppresses its ability to bind the poly (U) tract of the 3′ non-coding region of the genome of hepatitis C virus (HCV), which is required for efficient replication and/or translation (Tian and Klessig, unpublished results). It is interesting to note that glycyrrhizin, a compound derived from *Glycyrrhiza foetida* (common name licorice), also binds to human GAPDH and alters its activities much like SA – see below (14). Moreover, glycyrrhizin has anti-HCV activity and has been used for decades in Japan to treat chronic HCV infection (80, 81). Together, these findings suggest that SA or its more potent derivatives (see below) might be useful treatments for HCV infection.

In addition to GAPDH’s role in viral infection, it is a major suspect in several neurodegenerative diseases in humans, including Huntington’s, Parkinson’s, and Alzheimer’s diseases (88). The central role human GAPDH plays in neurodegeneration was established by the pioneering work of Ishitani and Chuang (89), and later by Snyder and coworkers (90). The latter study also provided evidence for a novel cell death cascade involving GAPDH, nitric oxide, and the E3 ubiquitin ligase called Seven *in absentia* homolog (Siah) (91). In brief, oxidative stress conditions can lead to elevated levels of nitric oxide, which cause S-nitrosylation of GAPDH’s catalytic cysteine 150. This inactivates GAPDH’s glycolytic activity and induces its interaction with Siah, whose nuclear localization signal enables the complex to enter the nucleus (91). Since the complex between Siah and GAPDH stabilizes this E3 ubiquitin ligase, turnover of Siah’s nuclear target proteins is increased, which in turn leads to cell death. Underscoring the significance of this GAPDH/Siah cell death cascade is the demonstration that the anti-Parkinson’s disease drug deprenyl, which reduces neuronal cell death in both *in vitro* and *in vivo* models, prevents S-nitrosylation of GAPDH, blocks the GAPDH–Siah interaction, and inhibits GAPDH nuclear translocation (90).

Using recombinant human GAPDH, we demonstrated that SA not only binds this protein but also suppresses its ability to translocate to the nucleus and induce cell death at low micromolar concentrations (14). Several natural and synthetic derivatives of SA that bind GAPDH more strongly than aspirin/SA also were identified; importantly, their greater binding affinity is correlated with enhanced inhibition of GAPDH’s nuclear translocation and cell death induction. The natural SA derivatives, called amorfrutins, are produced by *G. foetida*, while the synthetic derivative, acetyl 3-aminoethyl SA, was designed based on the structure of the amorfrutins, as well as the ability of other SA-like compounds to very tightly bind GAPDH and HMGB1, our other newly identified SA/aspirin target (15).

In parallel, our high-throughput screens used to identify human SABPs uncovered HMGB1. HMGB1 is the most abundant non-histone protein in the nucleus. It binds to the minor groove of DNA and plays a central role in condensing DNA, which affects nucleosome packing, transcription, and DNA replication, repair, and recombination. In addition, when HMGB1 is passively released to the extracellular milieu due to tissue damage or necrosis, it functions as a damage-associated molecular pattern (DAMP) to activate the innate immune system (92, 93). Extracellular HMGB1 triggers inflammation by recruiting immune-related cells involved in fighting infection and repairing damaged tissue. In addition, it stimulates these recruited immune-related cells to express genes encoding pro-inflammatory signaling proteins called cytokines. The resulting inflammation protects damaged tissue against infection and promotes healing. In some circumstances, however, inflammation is not properly controlled or it persists (non-resolved); this can contribute to the pathogenesis of many inflammation-associated diseases, such as arthritis, atherosclerosis, lupus, inflammatory bowel disorders, and sepsis, and certain cancers, such as colorectal and mesothelioma cancers.

We have discovered that SA binds to HMGB1, thereby blocking its pro-inflammatory activities (15). It does so at concentrations (low micromolar) far lower than those required to suppress the enzymatic activity of COX1 and COX2. Notably, we found that HMGB1 induces the expression of *Cox2*, as well as cytokine genes, and that low levels of SA suppress this induction. Thus, SA, such as aspirin, can suppress inflammatory responses mediated by COX2, but SA does so by inhibiting COX2 synthesis, rather than its activity. The discovery that HMGB1’s pro-inflammatory activities are inhibited by low levels of SA provides one likely explanation for the protective effects of low-dose aspirin usage.

Analyses of amorfrutin B1 and acetyl 3-aminoethyl SA revealed that they bind to HMGB1 in the same site as SA but do so with higher affinity. Similar to their greater potency in suppressing GAPDH activity, these compounds were 40- to 70-fold more effective than SA at inhibiting HMGB1’s pro-inflammatory activities. The existence of natural and synthetic SA derivatives that are even more potent than aspirin/SA at suppressing HMGB1’s and GAPDH’s disease-associated activities argues that there is significant potential for the development of SA-based drugs with improved efficacy and, possibly, fewer negative side effects.

All eukaryotic cells, including plants, have HMGB1-related proteins. *Arabidopsis* has eight HMGB-type proteins, including HMGB3, which is present in the cytoplasm and in the nucleus. Given that human HMGB1 is a prototypic DAMP in animals (92, 93) and that its DAMP activities are inhibited by SA binding (15), we asked whether *Arabidopsis* HMGB3 (i) functions as a DAMP, (ii) binds SA, and (iii) exhibits reduced DAMP activity following SA binding (44). We found that introduction of HMGB3 into the extracellular space (apoplast) induced innate immune responses, including callose deposition, MAPK activation, defense gene expression, and enhanced resistance to a necrotrophic fungal pathogen. Like its animal counterpart, HMGB3 bound SA and this binding suppressed its ability to induce innate immune responses and protect against pathogen infection.

## Why Might Animals have so Many SA Targets

Further research will likely uncover additional SA targets and help clarify which are responsible for SA’s beneficial therapeutic activity, as well as its negative side effects. The potentially large number of SA targets, combined with the multiple pharmacological effects mediated by SA and its prodrug aspirin, and the widespread use of aspirin and/or natural SA derivatives (which our studies suggest are the basis for at least some traditional medicines) suggest that much remains to be done in order to elucidate SA’s mechanisms of action. We predict that SA (and aspirin) exerts its effects in humans *via* multiple mechanisms of action that are mediated by a variety of targets. Such a scenario would be consistent with our discovery that plants contain more than two dozen proteins through which SA regulates immunity and other plant processes. The majority of animals eat plants, which exposes them to SA and its derivatives on a regular basis. Indeed, vegetarians contain similar levels of SA and its urinary metabolite salicyluric acid as individuals taking low-dose aspirin (94). However, dietary intake of SA appears to account for only a modest portion of the serum and urinary salicylates present in animals. Analyses of germ-free animals indicate that serum SA is not synthesized by gastrointestinal microbes. Rather, studies with ^13^C-labeled benzoic acid suggest that animals synthesize endogenous SA in large part using this precursor. Benzoic acid and its salts are found in high amounts in some fruits and vegetables, and thus it might contribute to the modest variability in serum SA associated with diet. Also, benzoic acid may be synthesized endogenously in animals using phenylalanine as a precursor. Based on these findings, Paterson and coworkers (94) suggested that it is “increasingly likely that SA is a biopharmaceutical with a central, broadly defensive role in animals as in plants.” Low levels of SA, resulting from dietary intake of SA and endogenous synthesis from benzoic acid/benzoate, might have led to the emergence of multiple SA targets in animals. If future studies confirm this hypothesis, it is highly likely that a variety of SA targets common to both kingdoms will be identified. Their characterization will not only help elucidate the mechanisms through which SA exerts its varied effects but also should provide clues for devising highly effective strategies to control pathological processes in plants and animals.

## Conclusion

Salicylic acid acts through many targets, rather than a few receptors, to mediate its many effects on diverse physiological and pathological processes in plants. The presence of SABPs exhibiting a wide range of affinities for SA, combined with the varying SA levels found in specific subcellular compartments, in different tissues, at different developmental stages, or during responses to environmental cues, provides tremendous flexibility and multiple mechanisms through which SA can exert its effects in plants. Animals have multiple targets of SA and its derivatives besides cyclooxygenases COX1 and COX2, which are the two major targets of aspirin. The discovery that HMGB1’s pro-inflammatory activities are inhibited by low levels of SA provides one likely explanation for the protective effects of low-dose aspirin usage. The existence of natural and synthetic SA derivatives that are even more potent than aspirin/SA at suppressing HMGB1’s and GAPDH’s disease-associated activities argues that there is significant potential for the development of SA-based drugs with improved efficacy and, possibly, fewer negative side effects. Low levels of SA, resulting from dietary intake of SA and endogenous synthesis from benzoic acid/benzoate, might have led to the emergence of multiple SA targets in animals, as in plants, some of which are common to both kingdoms.

## Author Contributions

DFK, MT, and HWC wrote the manuscript.

## Conflict of Interest Statement

The authors declare that the research was conducted in the absence of any commercial or financial relationships that could be construed as a potential conflict of interest.
